# 

**Published:** 1842-07-30

**Authors:** 


**CONTENTS OF No. 31.**

Dr. Harris' Case of Corneous Excrescence on the Leg, - - - - 481

Remarks by Dr. Reynell Coates, - - - - - - 482

BIBLIOGRAPHICAL NOTICE: Statistics of the results of the Capital Operations in the Hospitals of Paris. Of the Mortality after Amputations. By M. Malgaigne, Surgeon to Bicètre, - 483

ANALECTA: On the Treatment of Fracture of the Neck of the Thigh Bone, within the Capsular Ligament. By a Medical Officer of the Army, - - - - - - - - - - 490

Remarks by Dr. Reynell Coates, - - - - - - 493

Dr. Samuel S. Brame's Case of Artificial Premature Labour, 496

Spinal Distortions. Division of the Muscles of the Back, - 496

